# Pruritic Rash in a Patient with Hodgkin's Lymphoma

**DOI:** 10.7759/cureus.2450

**Published:** 2018-04-08

**Authors:** Audrey Le, Dimitrios Farmakiotis, John L Reagan

**Affiliations:** 1 Internal Medicine, Rhode Island Hospital, Alpert Medical School of Brown University; 2 Infectious Diseases, Rhode Island Hospital, Warren Alpert Medical School of Brown University; 3 Oncology-Hematology, Rhode Island Hospital, Warren Alpert Medical School of Brown University

**Keywords:** bleomycin-induced flagellate erythema, flagellate dermatosis, abvd, bleomycin, pruritic rash

## Abstract

Bleomycin-induced flagellate erythema (FE), a skin finding associated with cutaneous deposition of bleomycin, is so called due to its characteristic pattern of whip-like, linear streaks. As bleomycin use in standard chemotherapeutic regimens has decreased, the clinical diagnosis has become increasingly rare. The authors present a case of a 43-year-old female patient with Hodgkin’s lymphoma on her first cycle of adriamycin, bleomycin, vinblastine, and dacarbazine (ABVD) treatment, who subsequently developed a diffuse rash classic for FE. This benign condition is important to recognize to avoid potentially unnecessary and harmful treatment for other dermatologic diagnoses for which it may be mistaken. In severe cases of FE, discontinuation of bleomycin should be considered.

## Introduction

Bleomycin is a sulphur-containing antimicrobial derived from Streptomyces verticillus used in standard chemotherapy for Hodgkin's lymphoma, germ cell tumors, Kaposi sarcoma, and pleurodesis for malignant pleural effusions [1-4]. Bleomycin penetrates all tissues, but is quickly inactivated by bleomycin hydrolase, which is absent in the lungs and skin; thus its toxic manifestations occur predominantly in these two organs [1, 3, 5]. Studies show that 60-70% is excreted in the urine [3], hence patients with renal dysfunction may be at higher risk for toxicity [2, 6]. Bleomycin-induced flagellate erythema (FE), associated with skin deposition of bleomycin, is named after the initial eruption of erythematous linear streaks that appear ‘whip-like’ [3, 7].

We present a case of a female patient with Hodgkin’s lymphoma who presented with classic findings for FE after initiation of chemotherapy.

## Case presentation

**Figure 1:**
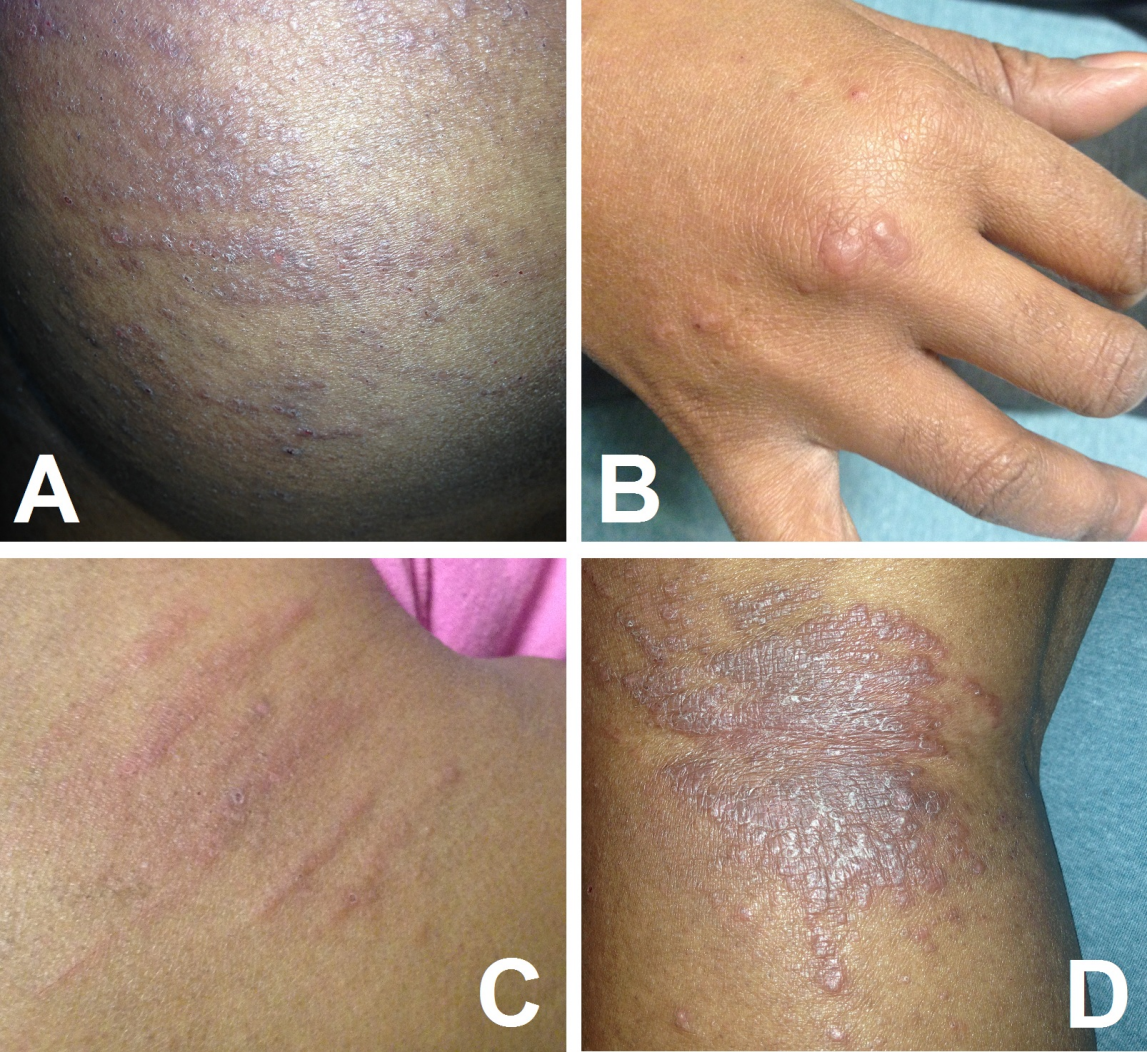
Bleomycin-induced flagellate erythema The pruritic maculopapular rash and nodules, on the torso (A), upper (B) and lower (C, D) extremities, with which our patient presented after her first cycle of ABVD, classic for bleomycin-induced flagellate erythema. ABVD - adriamycin, bleomycin, vinblastine, and dacarbazine.

A 43-year-old woman with classic Hodgkin’s lymphoma (HL) presented on Day 7 of her first cycle of adriamycin, bleomycin, vinblastine, and dacarbazine (ABVD) with a diffuse, pruritic maculopapular and nodular rash, which had started from her knuckles and progressed to the torso and lower extremities (Figure 1).

The presence of her diffuse rash along with a pulmonary cavitary lesion prompted infectious workup. She had a positive urine histoplasma antigen (Quest diagnostics, NJ, USA) and was admitted to the hospital for treatment of presumed histoplasmosis with intravenous amphotericin-B. Skin biopsy was performed and histopathology showed inflammatory changes suggestive of drug reaction. Repeat histoplasma urinary antigen was negative, and computerized tomography of the chest showed significant decrease in the size of the pulmonary lesion after one ABVD cycle, consistent with HL as the cause of her pulmonary cavitary lesion. She was discharged with a diagnoses of bleomycin-induced flagellate erythema and falsely positive urine histoplasma antigen. Her skin lesions completely resolved over the next two weeks with symptomatic treatment (two antihistamines for intense pruritus, triamcinolone 0.1% cream, and petrolatum-based skin protectant).

## Discussion

FE has become increasingly rare as bleomycin use has decreased; however, it is important to note that there have been reported cases of FE amongst other patient populations, particularly those with dermatomyositis and adult-onset Still’s disease, as well as in individuals after shiitake mushroom ingestion [6-8]. Peplomycin, a bleomycin analogue used in the treatment of prostate cancer, breast cancer, and HL, can also cause FE [7, 8]. It is important to rule out other causes of parallel, streak-like skin lesions, such as Koebner phenomenon due to inflammatory, infectious, or self-inflicted processes, or photodermatitis [7]. Bleomycin-induced FE has variable onset, occurring anywhere between several hours to two months after drug administration [9]. There is no clear association between FE and the route of drug administration, dose, or type of underlying disease being treated [6]. Lesions most frequently appear on the torso, extremities, and nape of the neck, but they can present on the face and scalp as well  [4, 5, 7, 9]. FE requires symptom relief with antihistamines and corticosteroids (topical or oral). Discontinuation of bleomycin is generally reserved for severe cases of skin toxicity, since in an open-label, randomized, multicenter trial comparing ABVD with regimens omitting bleomycin, dacarbazine, or both, in early-stage favorable Hodgkin’s lymphoma, there was no significant difference in survival. However, patients treated without bleomycin had higher rates of relapse and worse control of tumor burden [10].

Given the severity of symptoms in our patient, she resumed AVD chemotherapy (without bleomycin), followed by salvage brentuximab and bendamustine for primary refractory disease, and subsequent autologous stem cell transplantation with post-transplant brentuximab. She is disease-free and doing well two years after FE diagnosis, 18 months after transplant.

## Conclusions

Clinicians should be aware of this uncommon, yet benign complication, to avoid unnecessary workup and potentially harmful empiric treatment for other causes of skin lesions in immunocompromised patients given the broad differential diagnosis. In severe cases of FE, discontinuation of bleomycin should be considered.
